# Traits Expansion and Storage of Soybean Phenotypic Data in Computer Vision-Based Test

**DOI:** 10.3389/fpls.2022.832592

**Published:** 2022-03-01

**Authors:** Yongchao Xing, Peixin Lv, Hong He, Jiantian Leng, Hui Yu, Xianzhong Feng

**Affiliations:** ^1^School of Mechanical, Electrical and Information Engineering, Shandong University, Weihai, China; ^2^Northeast Institute of Geography and Agroecology, Chinese Academy of Sciences, Harbin, China

**Keywords:** soybean test, phenotypic data, computer vision, traits expansion and storage, soybean AI breeding platform

## Abstract

Phenotypic traits of crops are an important basis for cultivating new crop varieties. Breeding experts expect to use artificial intelligence (AI) technology and obtain many accurate phenotypic data at a lower cost for the design of breeding programs. Computer vision (CV) has a higher resolution than human vision and has the potential to achieve large-scale, low-cost, and accurate analysis and identification of crop phenotypes. The existing criteria for investigating phenotypic traits are oriented to artificial species examination, among these are a few traits type that cannot meet the needs of machine learning even if the data are complete. Therefore, the research starts from the need to collect phenotypic data based on CV technology to expand, respectively, the four types of traits in the “Guide to Plant Variety Specificity, Consistency and Stability Testing: Soybean”: main agronomic traits in field investigation, main agronomic traits in the indoor survey, resistance traits, and soybean seed phenotypic traits. This paper expounds on the role of the newly added phenotypic traits and shows the necessity of adding them with some instances. The expanded traits are important additions and improvements to the existing criteria. Databases containing expanded traits are important sources of data for Soybean AI Breeding Platforms. They are necessary to provide convenience for deep learning and support the experts to design accurate breeding programs.

## Introduction

In recent years, artificial intelligence (AI) technology has been widely used in the field of agronomy, which is used to image classification, object recognition, and feature extraction (Pound et al., 2018). The difficulty of plant science research is gradually changing from gene analysis to phenotypic analysis (Mir et al., 2019). The correlation analysis between Phenomics and other omics can analyze the biological laws of crops and effectively serve agricultural production (Zhou et al., 2018). Hu et al. (2019) explains the development of plant phenotypes, and explains in detail the importance of phenotypic collection techniques and image data analysis methods to promote plant phenotype research; Ma (2020) uses computer vision (CV) technology to extract the phenotypic traits of soybean seeds; Ren et al. (2020) proposes a model named Deconvolution-GuidedVGGNet to realize plant leaf disease species identification and spot segmentation at the same time; Guo et al. (2021) use the improved yolov4 to detect the pods of soybean plants.

However, soybean has relatively few phenotypic traits. In the preliminary study of phenotypic traits of soybean varieties using CV, only the plant height and the phenotypic traits of leaf parts are extracted. The preliminary study could not provide enough data to meet the needs of AI breeding. In this study, phenotypic traits of soybean at various stages are expanded based on “Guide to Plant Variety Specificity, Consistency and Stability Testing: Soybean” (Ministry of Agriculture and Rural Affairs of the People’s Republic of China, 2018), using CV processing methods and AI technology, a sufficiently large amount of data is obtained to establish a quantitative analysis technology for the main agronomic traits of different soybean varieties in the field investigation, indoor test, disease resistance, and quality.

## Quantitative Analysis of Soybean Field Survey Traits Based on Computer Vision Technology

**FIGURE 1:**
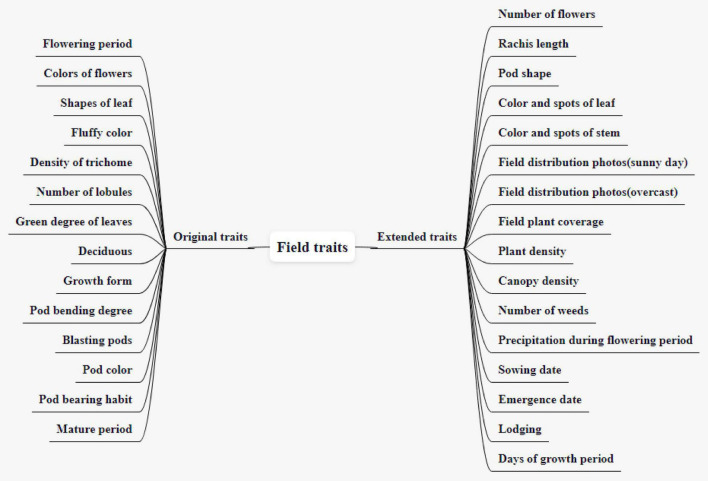
Field plant traits and the expansion.

The CV-based phenotypic data acquisition methods could find and remember more details than that were seen to the naked eye, so the currently common 14 field traits are expanded to 21 different traits, as shown in Figure 1.

### Common Field Investigation Traits

There are 14 original field traits, including flowering period, colors of flowers, shapes of leaf, fluffy color, the density of trichome, number of lobules, green degree of leaves, deciduous, growth form, pod bending degree, blasting pods, pod color, pod bearing habit, and mature period. The sowing date refers to the date on the day of sowing, expressed as month-day. The flowering period refers to the date when 50% of the plants begin to bloom, expressed as month-day. The colors of flowers refer to the color of petals, divided into white and purple. Shapes of the leaf refer to the shape of the middle leaf of the three mature compound leaves in the middle and upper part of the investigated plant during the peak flowering period. It is divided into four types: needle-shaped, triangular, apical ovate, and round ovate (In the north, it is generally pointed leaves, while in the south, it is generally circular leaves. In the north, the sunshine is short, and the light is relatively insufficient, thus, avoid shielding). The fluffy color refers to the color of the fluff on the upper part of the stem or the pod skin of the investigated plant when it is mature, divided into gray and brown (The fluffy color is generally gray in the northeast and brown in the south). The density of fine hair is divided into nine levels, which are extremely thin, extremely thin to thin, thin, thin to medium, medium, medium to dense, dense, dense to extremely dense, and extremely dense. The number of lobules is divided into three lobules, five lobules, and many lobules. The green degree of leaves can be divided into nine types: very light, very light to light, light, light to medium, medium, medium to deep, deep, deep to very deep, very deep. There are three kinds of deciduous traits: non-deciduous, semi-deciduous, and deciduous. According to the growth form of the main stem, it can be divided into semi-straggling, straggling, semi-erect, and erect, which can be used to establish the prediction model of yield per unit area. Pod bending degrees can be divided into four types: none or very weak, weak, medium, and strong. There are 9 kinds of blasting pods: none or very light, none or very light to light, light, light to medium, medium, medium to heavy, heavy, heavy to very heavy, and very heavy. Pod color is the ripening pod color, divided into white yellow, light yellow, light brown, medium brown, dark brown, and black, which is a total of six colors. Pod-bearing habits are divided into infinite, finite, and sub-finite. Among them, the infinite pod-bearing habit means that new leaves can be produced at the top of the main stem when it blooms. The flowering and pod-setting sequence is from bottom to top, with small top leaves, short inflorescences, scattered pods, and generally 1–2 pods at the top of the main stem. Finite pod-bearing habit means that no new leaves appear on the main stem when it blooms, and there are obvious inflorescences at the top and pod-bearing clusters. The sub-finite pod-setting habit means that the growth traits and pod-setting status at the top of the main stem are between infinite and finite. Generally, 3–4 pods grow on the top of the main stem. The mature period refers to the date when 95% of the pods of the whole plant become mature color, and the plants that start to make a noise when shaking reach more than 50%, expressed in month–day.

The device for acquiring soybean field images is DJI Phantom 4Pro, which has strong battery life, a 1-inch, 20-megapixel sensor, and a dynamic range close to 12 stops. It has better image quality in terms of the level of detail and low-light conditions. Adapt to the changing light intensity in the field. The colors of flowers and shapes of leaves in the original survey standard can only be observed by naked eyes, and the flower color can only be divided into white and purple. To record the flower color more accurately and specifically, it is necessary to use CV technology to process the collected images to obtain the average RGB value of the flower. The RGB is the most commonly used color space, consisting of three-color channels: red (R), green (G), and blue (B). Soybean images in the field under natural conditions are easily affected by light and obstructions and are very sensitive to brightness. In the RGB color space, these three components are highly correlated. The change of brightness will cause the three components to change accordingly, and the naked eye has different perceptions of the three colors. In the HSV color space, H refers to hue, S refers to saturation, and V refers to a degree. It is closer to people’s experience of color perception than RGB, more intuitively expresses the hue, vividness, and brightness of colors, and is more convenient for color contrast. Therefore, in image processing, first, convert the color space of field soybean image from RGB color space to HSV color space, and then, define the range of colors of flowers in HSV color space, generate a mask according to the threshold, and perform bitwise and operation between the mask and the original image. The flower part is extracted from the image, and finally, the color space is converted to RGB, and the RGB average value of the non-zero pixels is extracted as the colors of flowers. The shapes of the leaves are obtained after the edge detection using the Laplacian of the Gaussian operator. The Laplacian of Gaussian is composed of Gaussian filtering and Laplacian. It is an edge detection operator based on the second-order differential method. The Laplacian operator can highlight the areas where the intensity changes rapidly in the image, so, it is often used in edge detection. Since the Laplacian operator is more sensitive to discrete points and noise, it is necessary to use a Gaussian smoothing filter to smooth the image before performing the Laplacian operation to reduce the sensitivity of the Laplacian operation to noise and improve the robustness to noise and discrete points. The following are the Algorithm steps: ➀Use Gaussian smoothing filter for smoothing; ➁Use Laplacian to calculate the second derivative; ➂Detect the zero-crossing point in the image; and ➃Set the zero-crossing point as the threshold, and only keep those strong crossing points (there is a big gap between the positive maximum and the negative minimum).

### Field Investigation Traits Extended by Computer Vision Techniques

**FIGURE 2:**
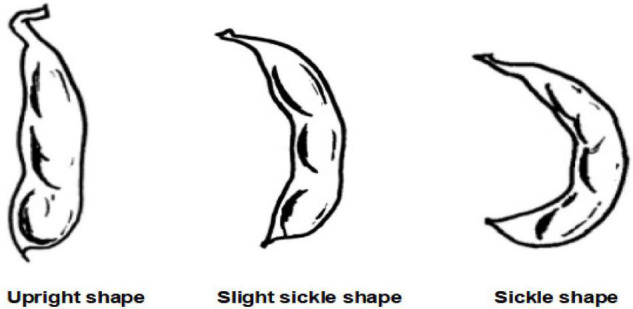
Pod shapes.

The extended traits of field traits include sowing date, emergence date, lodging, days of the growth period, number of flowers, rachis length, pod shape, color and spots of leaf, color, and spots of the stem, field distribution photographs (sunny day), field distribution photographs (overcast), field plant coverage, plant density, canopy density, number of weeds, and precipitation during the flowering period. At present, there are 16 extended traits. The sowing date and emergence date are expressed in month-day. Lodging is divided into 5 grades according to the lodging rate. Grade 1 means not falling, and all plants stand upright; Grade 2 means light fall, 0 < lodging plant rate ≤ 25%; Grade 3 means mid-lodging, 25% < lodging plant rate ≤ 50%; Grade 4 refers to heavy lodging, 50% < lodging plant rate ≤ 75%; and Grade 5 refers to severe lodging, lodging plant rate > 75%. Days of growth period refers to the total number of days from the emergence date to the mature period, expressed in days. The number of flowers refers to the total number of flowers on the flower axis. The purpose of expanding this trait is to establish a relationship with varieties. The rachis length is used to measure the number of flowers. Those with a short axis have fewer flowers and those with a long axis have more flowers. As shown in Figure 2, the pod shape is divided into straight shape, slightly curved sickle shape, and curved sickle shape, which are used to correlate with varieties and establish a yield prediction model. The mature color of the pod is the color of the pod when it is mature. It can be divided into five colors: Grass yellow, gray-brown, brown, dark brown, and black. The growth morphology can be divided into vine type, semi-erect type, and erect type according to the growth morphology of the main stem, which is used to establish the prediction model of unit yield. The color and spots of the leaf and the color and spots of the stem are used to establish the early warning model of diseases and pests. Both aforementioned extended traits can be represented in the form of pictures. The specific network can be trained and tuned according to the acquired soybean leaf disease and insect image to achieve high leaf disease and pests’ recognition accuracy. A convolution neural network was used to extract image features of four kinds of disease spots, and a support vector machine model for disease recognition was established (Qin et al., 2017). The retrieval and analysis of three soybean leaf diseases are realized by using the color, shape, and texture characteristics of images (Jayamala et al., 2016). Xu et al. (2020) uses the acquired small data samples of corn (Zea mays) leaf diseases and insect pests’ images, a convolutional neural network based on transfer learning is proposed to identify corn diseases. The data set is expanded by data enhancement operations such as rotating and folding the original image. The model is improved based on the VGG-16 model. After comparing different migration learning training mechanisms and tuning the model, the average recognition accuracy of pests and diseases on the test set is as high as 95.33%. A trait feature descriptor-chord feature matrix is applied to the classification of soybean leaf images, which can distinguish the leaves of different soybean varieties, and is used to establish the relationship between varieties and leaf diseases and insect pests (Wang et al., 2017). Diseases can also develop on the stems of soybean plants. For example, soybean stem blight first occurs in the lower part of the stem, and then, gradually spreads to the upper part of the stem. In the early stage of the disease, the stems produce long oval lesions, which are grayish-brown, and then gradually expand into long black strips. After the leaves are fallen, the symptoms are more obvious on the stems of the plants before harvest, forming patches of oblong diseased spots. Therefore, images of soybean stems are collected to establish early warning models of diseases and insect pests, such as stem blight.

We use Dajiang UAV (DJI UAV) to capture the field distribution photographs on a sunny day and overcast and use CV technology to compare and process the acquired images of a sunny day and overcast, which can eliminate the influence of weather and obtain the traits of each plant such as plant height. These traits can be used to establish a yield forecast model. The DJI Matrice 600Pro UAV is used for field shooting, which is equipped with an RGB camera that is a digital camera, a near-infrared multi-spectral camera, and an infrared thermal imager to form the UAV imaging system (Zhou et al., 2020). They obtained field data of 116 soybean genotypes, and obtained 7 image features through image processing technology, including temperature, hue, color saturation, canopy size, and plant height. A support vector machine model is developed, and the degree of soybean canopy withering is graded and is scored according to the acquired image features. Studies have shown that UAV-based image technology has great potential in selecting drought-tolerant soybeans. Therefore, obtaining field distribution photographs can extract the traits of each plant, which plays an important role in the subsequent establishment of a yield prediction model and the selection of drought-tolerant varieties. Field plant coverage is obtained by image processing on the original map of field distribution, which is used to establish relationships with other traits. The plant density is also obtained by processing field images, and the plant density is used to analyze the suitable density of soybean growth. Canopy density refers to the ratio of the total projected area of the canopy on the ground under direct sunlight (canopy width) to the total area of the ground, and it is used to reflect the density of planting. The number of weeds refers to the percentage of weeds in the number of plants, which is used to analyze the appropriate density of soybean growth.

The principle of obtaining the color of the leaf surface and the surface of the plant stem is the same as that of the flower color. As mentioned above, the colors and spots on the surface of leaves and plant stems are used to establish warning models of diseases and insect pests, so, the first thing to do is to mark the leaves with spots. The spots are divided into soybean downy mildew spots and soybean gray spot spots, soybean root rot spots, soybean brown spots, soybean bacterial spots, premature aging spots, etc. Then, use the marked image as a training set for the training of the target detection model. The purpose of target detection is to detect the spots in the new image and recognition.

Target detection technology, based on deep convolutional neural networks, has achieved good results in many fields (Zhang et al., 2019). The target detection method used in this study is YOLOV4 of the YOLO series. The network is mainly composed of four parts: input, Backbone, Neck, and prediction part. At the input end, Mosaic data enhancement methods are adopted to enrich the data set, which can adapt anchor calculation and image scaling. Mosaic data enhancement is a data enhancement method that takes four photographs and splices them in the way of random scaling, clipping, and arrangement. The backbone part refers to the convolutional neural network that aggregates features on different image granularity, while the neck part refers to a series of network layers with combined features, which finally transmits the feature map to the prediction layer, lastly, the head part refers to predicting the feature map, generating the prediction boxes, and giving the label.

## Quantitative Analysis of Soybean Seed Traits in Indoor Test Based on Computer Vision

**FIGURE 3:**
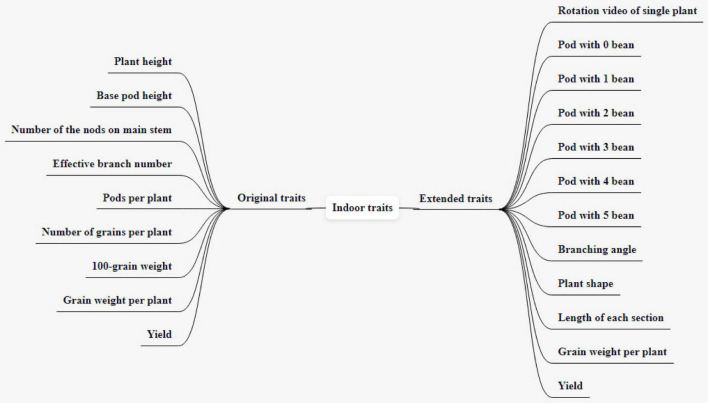
Indoor traits and the expansion.

Indoor traits include traits directly obtained through relevant simple operations indoors and traits obtained by 360° rotating video shooting of soybean plants fixedly harvested through specific devices, and processing pictures of some frames through CV technology. Indoor traits include seven original traits and 12 expanded traits, as shown in Figure 3.

### Original Indoor Traits

The original traits include plant height, base pod height, number of the nodes on the main stem, effective branch number, pods per plant, number of grains per plant, 100-grain weight, a total of seven traits. Plant height refers to the height from cotyledon node to plant top (including top inflorescence), expressed in cm, accurate to 0.1 cm, and the average value is taken for 10 consecutive plants. Base pod height refers to the height from the cotyledon node to the lowest pod insertion position, expressed in cm, the average value of 10 consecutive plants. The number of nodes on the main stem refers to the actual number of nodes at the top of the main stem (excluding the top inflorescence), which is related to the lodging resistance of the plant. The effective branch number refers to the number of branches with more than one pod, and the branches have at least two nodes. Excluding the secondary branches, take the average value of 10 consecutive plants. The number of pods per plant refers to the number of pods containing more than one full seed on a plant. The number of grains per plant refers to the number of grains obtained by one plant, including all intact grains, immature grains, insect eroded grains, and diseased grains. The 100-grain weight is obtained by randomly taking 100 grains from the intact grains of the sample 2 times, weighing them separately, and calculating the average value (if the difference between the two weighting values exceeds 0.5 g, resample and weigh), the unit is expressed in g. Soybean plants are fixed by a specific device, a soybean plant phenotype measuring instrument, and a background plate is installed to fix the shadow-absorbing screen to ensure the image quality. Indoor images of individual plants are obtained by the camera (BasleracA4112-20uc), with a resolution of 4,096px*3,000px. The camera parameter configuration is shown in Table 1. The background of the image is consistent with the lighting environment, and only color homogenization is required to deal with the slight background chromatic aberration. Both the plant height and the base pod height can be obtained by processing the image of the plant by CV technology. Compared with manual measurement, the error is within 1 cm. A fixed-length reference object is given next to the plant, and the plant and the reference object are taken together when the image is collected. We convert the RGB image to a grayscale image, set the grayscale value of the measurement target and the reference object to 1, and set the grayscale value of the rest to 0. We obtain the number of pixels of the target and reference, calculate the ratio of the number of pixels of the target to the number of pixels of the reference, and multiply the actual length of the reference with this ratio to get the actual length of the target. The number of the nodes on the main stem is obtained by calculating the number of joints of each node on the stem, and the gray image of the plant contour is obtained by edge detection technology. After eliminating the interference of non-branch contours, the effective branch number can be obtained by calculating the number of branch contours. The correct result rate of the number of the nodes on the main stem and effective branch number can reach 96%, which is slightly lower than that of naked eye recognition, but it greatly shortens the measurement time.

**TABLE 1:** Basler acA4112-20uc camera parameters.

Parameters	Value
Pixel format	Bayer RG 8
Exposure auto	Off
Exposure time (μs)	3,000
Gain auto	Off
Gain (dB)	0
Balance white auto	Continuous
Acquisition burst frame count	80
Trigger selector	Frame burst start
Trigger mode	On
Trigger delay (μs)	500,000

### Indoor Investigation Traits Extended by Computer Vision Techniques

**FIGURE 4:**
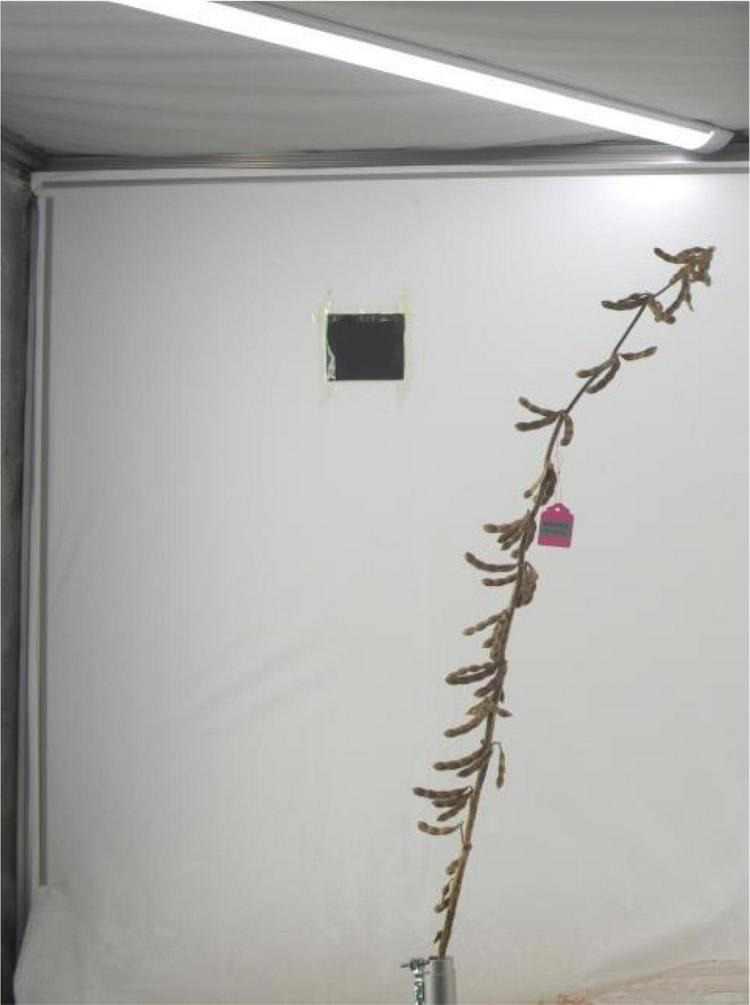
Single plant image.

**FIGURE 5:**
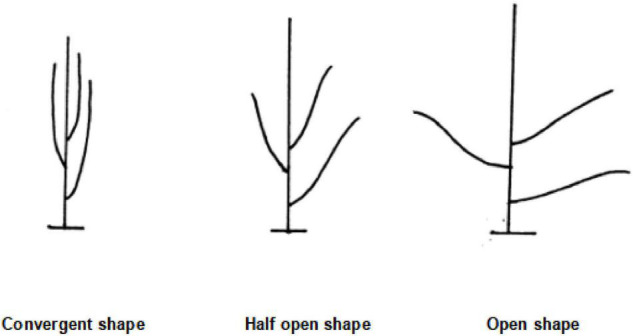
Three plant shapes.

The expanded traits include grain weight per plant, yield, rotation video of a single plant, a pod with 0 beans, a pod with 1 bean, a pod with 2 beans, a pod with 3 beans, a pod with 4 beans, a pod with 5 beans, branching angle, plant shape, and length of each section, totaling 12 traits. The grain weight per plant refers to the grain weight of the sample (including immature, worm-eaten, and diseased grains) (g/plant). Yield is expressed in grams (g) and kept in whole numbers; the sun-dried seeds are weighed after the moisture content reaches below 13% and impurities are removed. Rotation video of a single plant refers to the video taken by a single plant rotating one circle on a mechanical device (soybean plant phenotype measuring instrument). The steps of the original soybean plant rotation video preprocessing method are as follows: to obtain each frame in the double plant rotation video, cut it into a single plant image by using the image clipping algorithm, then save it. The pixel of the single plant image is 2,048 * 3,000. Figure 4 shows the input single plant image. We capture images of certain frames in the video and use them as the original data for subsequent target detection. The background of these images is the same as the lighting environment, and no preprocessing is required. The 0–5 pods in the intercepted image were labeled, and appropriately improved the target detection algorithm YOLO V4 mentioned above. After the last layer of the model backbone network, the global attention module is introduced. The function of this module is the maximum pooling and average pooling of the global channel information are used to obtain new weights, and the re-weighting calculation generates the output of the module. The improved method extracts the protruding part of the pod and the edge of the pod as key features, which improves the ability of the traits of the model. After tuning and training the improved YOLO V4, the target detection model is obtained. The evaluation indicators of the model are shown in Table 2. The average accuracy of this model is 7% higher than that of YOLO V4, reaching the accuracy of manual identification, but the identification speed is up to 240 plants/hour, which is significantly improved compared with manual identification. The model can detect the pods on the plant and classify the pods belonging to several pods. All the images are input into the trained target detection model for detection, and the data of 5 traits of 0–5 pods are obtained. According to the number of pods from 0 to 5, the number of pods per plant and the number of grains per plant can be calculated in common indoor copying traits. The number of pods from 0 to 5 is used to establish the relationship between variety and yield, and to establish a yield prediction model. Uzal et al. (2018) uses convolutional neural network CNN to classify pods with different numbers and confirms that CNN is significantly better than the classical method based on pre-designed feature extraction. The original image of this method must be a single-pod image of soybeans, and the Yolo model we used is less restrictive to the original image. It is not necessary to remove the pods from the plants to shoot, just select some frames of images from the video taken by rotating the whole plant 360 degrees. The branching angle, plant shape, and length of each section are all obtained by image processing. The branching angle refers to the angle between the main stem and the branch, which is used to judge the shape of the plant. According to the branch angle, the plant shape is divided into three types: convergent, semi-opened, and opened (Figure 5). The convergent shape refers to the small angle between the lower part of the branch and the main stem, and the upper and lower branches are compact; the open shape refers to the large branch angle, and the upper and lower branches are loose; the semi-open shape is between convergent and open. The length of each node and the number of main stem nodes in the original traits are obtained through image processing technology, with cm as the unit. The error between the calculated value of each section length and the actual measured value is within 0.2 cm. The recognition accuracy of the number of main stem nodes is as high as 98%. The branching angle, plant shape, and length of each section are used to establish contact with the variety.

**TABLE 2:** Model evaluation index.

Model	Average precision (%)						Mean average precision (%)
	
	0 bean	1 bean	2 beans	3 beans	4 beans	5 beans	
Manual measurement	–	–	–	–	–	–	84
YOLOV4	79.40	85.81	90.37	96.36	97.20	18.51	77.94
Improved YOLOV4	89.26	98.00	99.83	98.26	98.98	21.91	84.37

### Quantitative Analysis of Disease Resistance Traits Based on Computer Vision

The traditional investigation of disease resistance traits generally adopts the method of artificial inoculation identification, which requires a lot of manpower and material resources, and the cultivation cycle is long. How to combine technology with the prevention and control of diseases and insect pests is one of the important goals of achieving breeding. One of the goals. At present, there are more than 120 kinds of soybean pests and diseases reported in the world, and 52 kinds of soybean pests have been reported in my country. Based on the research background and existing experimental results, this study only focuses on the soybean diseases and pests that are more harmful in Northeast China. The original disease resistance traits are only gray spot disease and virus disease, so, it is expanded and increased on this basis.

According to the systematic investigation and research conducted by scientific researchers in 2015, the following diseases are common, and they are arranged according to the incidence degree of each disease (Zhu, 2014; Li et al., 2016; Liu, 2017; Gong, 2019).

### Fungal Diseases

Types: (1) soybean root rot disease; (2) soybean downy mildew disease; (3) soybean brown stripe disease; (4) soybean brown spot disease; (5) soybean gray spot disease; (6) soybean stem blight disease; (7) soybean pseudo stem mildew stem blight disease; (8) soybean anthracnose disease; (9) soybean black spot disease; (10) soybean gray spot disease; (11) soybean damping-off; (12) soybean sclerotia disease; (13) soybean wheel blight; (14) soybean vertical blight; (15) soybean sandwich blight; and (16) soybean purple spot.

Grades: Grade 0: the whole plant leaves are free of disease; Grade 1: partial disease, with less than five disease spots; Grade 2: a small number of diseased spots in the whole area, the distribution area of diseased spots accounted for less than 1/4 of the whole area; Grade 3: most plants in the whole region are infected, and the distribution area of disease spots accounted for half of the whole area; Grade 4: diseased spots are common in the whole area, and a few plants die early due to disease; and Grade 5: there are a lot of disease spots in plants in the whole area, and most plants die early due to disease.

### Bacterial Diseases

Types :(1) soybean bacterial keratosis; and (2) soybean bacterial speckle.

Grade: Standard, refer to fungal diseases.

### Viral Diseases

Types: Soybean Mosaic virus disease.

Grades: Grade 0: the leaves are flat in the whole area, without any signs of disease; Grade 1:10% of plants in the whole region have 1–2 layers of upper leaf shrinkage; Grade 2:20–40% of plants have withered upper leaves; Grade 3: more than 50% plants in the whole region have severe leaf shrinkage, yellow spots, affecting growth; and Grade 4: more than 70% of plants in the whole region have severe leaf shrinkage and bud dead branches.

### Insect Pests

Types:(1) soybean cyst nematodes; (2) soybean heartworm; (3) soybean aphid; (4) soybean red spider; and (5) soybean meadow borer.

Grade: 0: the whole plant is free of disease; Grade 1: some leaves are infected, and the number of wormholes in infected leaves is less than 5; Level 2: a small number of plants in the whole region had wormholes in their leaves, and the distribution area of wormholes accounted for less than 1/4 of the leaf area; Level 3: most leaves of plants in the whole region have wormholes, and the distribution area of wormholes accounts for half of the leaf area; Level 4: wormholes are common in plant leaves, and a few leaves died early because of wormholes; and Level 5: there are many wormholes in plant leaves in the whole area, and most leaves die early because of wormholes.

Techniques can be used when investigating disease resistance traits. In the analysis stage, the target detection model can be established through computer image processing, and the area suffering from the disease can be successfully divided, and then, real-time monitoring should be conducted. The degree of disease can also be classified according to the results of image processing, and different prevention and control measures can be adopted according to different grades.

**FIGURE 6:**
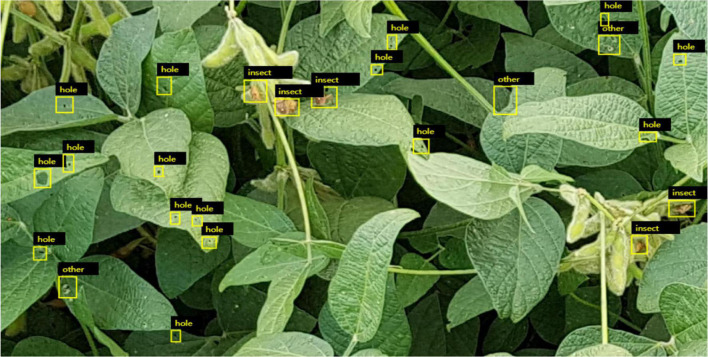
Preliminary labeling pictures of leaf diseases and insect pests.

In this study, pictures of soybean in the field have been collected by UAV, and more than 2,000 pictures have been labeled to make data sets. The labeled data sets are shown in Figure 6. The target category was preliminarily divided into three categories, namely, wormhole, pest, and other disease spots. The YoloV5 model was trained for detection and recognition, and the recognition rates of all three categories reached 95%. Among them, the target objects identified as pests can be further identified in the subsequent stage, and other disease spots can also be further identified to analyze which kind of disease spots.

## Quantitative Analysis of Seed Traits Based on Computer Vision

The selection of soybean seeds is the most important step in seed examination. To obtain soybean seed phenotypes for machine seed selection by CV, we added soybean seed test traits as an important extension of the standard. We expanded the original soybean seed test traits. Soybean seed test traits include 10 original traits and 18 expanded traits, a total of 28 traits.

### Original Soybean Test Traits

**FIGURE 7:**
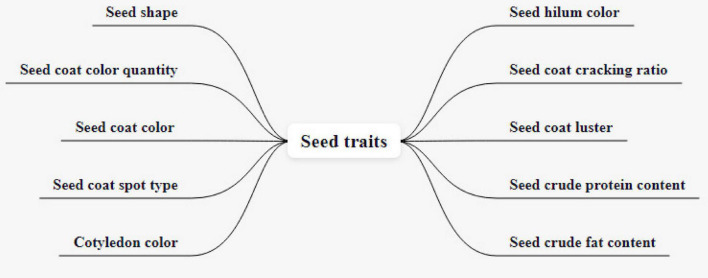
Soybean test original traits.

The original traits of the soybean test include seed shape, seed coat color quantity, seed coat color, seed coat spot type, cotyledon color, seed hilum color, seed coat cracking ratio, seed coat luster, seed crude protein content, seed crude fat content, a total of 10 traits, as shown in Figure 7. Seed shape is divided into five grades, which are spherical, elliptic, long elliptic, oblong-elliptic, kidney-shaped. The number of seed coat colors can be divided into monochrome and bicolor. Seed coat color is divided into white yellow, light yellow, yellow, yellow-green, green, light brown, brown, and black, a total of eight colors. The types of seed coat spots include tiger, saddle, and others. Midnight has three colors: yellow, chartreuse, and green. Umbilicus color is divided into light yellow, yellow, light brown, brown, light black, and black, a total of six colors. The seed coat cracking ratio is divided into nine grades: none or very low, none or very low to low, low to medium, medium to high, high to very high, and very high. Seed coat gloss is divided into none or none. The crude protein and crude fat content of seeds can be divided into 9 kinds: very low, very low to low, low, low to medium, medium, medium to high, high, high to very high, very high.

### Soybean Test Traits Extended by Computer Vision Techniques

**FIGURE 8:**
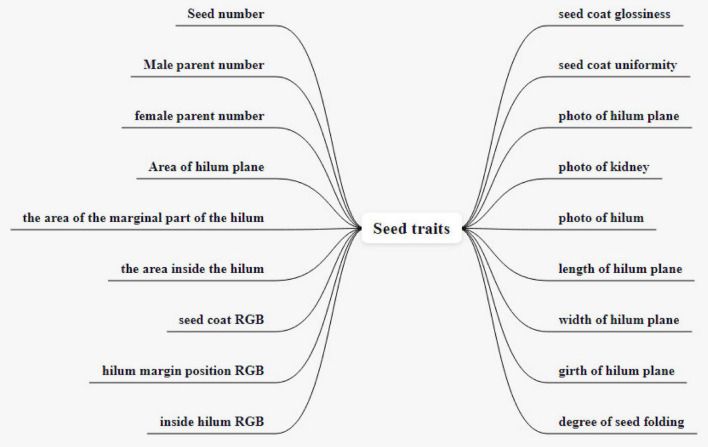
Soybean test expanded traits.

Soybean seed test traits are completely extended traits. Including seed number, male parent number, female parent number, area of hilum plane, the area of the marginal part of the hilum, the area inside the hilum, seed coat RGB, hilum margin position RGB, inside hilum RGB, seed coat glossiness, seed coat uniformity, photograph of hilum plane, photograph of kidney, photograph of hilum, length of hilum plane, width of hilum plane, girth of hilum plane, and degree of seed folding. There are 18 extended traits, as shown in Figure 8. It should be noted that the photographs of the hilum plane, kidney, and hilum may contain unextracted data, so, the three kinds of images are also represented as traits.

Seed number, male parent number, and female parent number are used to establish associations between phenotypic and genomic data and to establish a soybean knowledge graph. The original color traits are divided according to grades, and the expression is not accurate enough. So, we use RGB to represent color in extended traits. Seed coat RGB refers to seed coat color, used for color contrast between seed coats of different soybean varieties. The value range of seed coat glossiness is 0–1, used to describe the ability of seed coat to reflect light, replaced by the V of HSV space. The seed coat uniformity ranges from 0 to 1 and is used to describe whether the brightness distribution of the seed coat is uniform from center to edge. The area of the hilum plane refers to the surface where the umbilicus is located and the area of the part without the umbilicus, in mm^2^. The area of the marginal part of the hilum refers to the area of hilum, it includes the hilum margin (kidney), in mm^2^. The area inside the hilum refers to the surface on which the hilum is located; the area inside the hilum (hilum) is in mm^2^. Hilum margin position RGB refers to the color of the hilum margin (kidney) and is used to compare the color contrast of the hilum margin among different varieties. Inside hilum RGB refers to the color inside the hilum (hilum) and is used to compare the color inside the navel between different varieties. The photographs of the hilum plane are used to extract the features of the umbilicus. Photographs of the kidney is a seed hilum edge image extracted from the photographs of the hilum plane to extract seed kidney features. The photographs of the hilum are the internal images extracted from the photographs of the hilum plane and used to extract the traits of the hilum.

The hilum and kidney of seeds carry important genetic information. The area data of the above three parts can be used to establish the relationship between varieties and the prediction model of yield per unit area. The color traits of hilum are represented by RGB three-channel values and the values of a single channel range from 0 to 255. Values of different channels are separated by commas. The hilum and kidney of seeds carry important genetic information. The area data of the above three parts can be used to establish the relationship between varieties and the prediction model of yield per unit area. The color traits of hilum are represented by RGB three-channel values and the values of a single-channel range from 0 to 255. Values of different channels are separated by commas.

**FIGURE 9:**
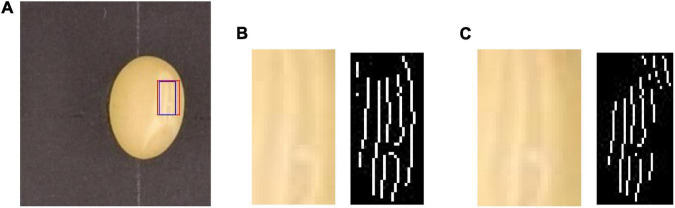
Photograph of a soybean seed **(A)** and umbilicus **(B)** and seed kidney **(C)** obtained using CV techniques.

These traits, which carry genetic information, are delicate but important to breeders. The length, width, girth, and area of the hilum are used for comparison among different varieties, and the unit is expressed in pixels. Figure 9 is a sample umbilical photograph from which we have extracted traits.

**FIGURE 10:**
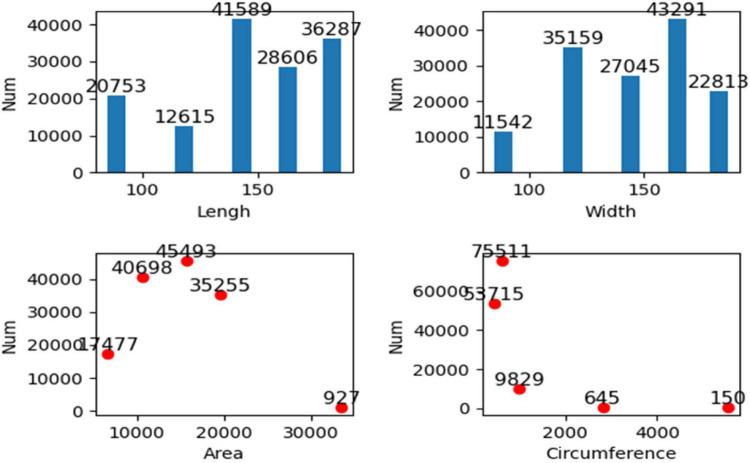
Cluster analysis of length, width, perimeter, and area.

We conducted k-means clustering analysis on the data of the above four traits of 139,850 seeds extracted so far, which are divided into five categories as the standard, and the results are shown in Figure 10. Figure 10 shows the corresponding quantities of five classes of the four traits. The five cluster centers of the length of the hilum plane are 90, 120, 146, 165, and 183, respectively, and it could be seen from the figure that most of the values of length of the hilum plane are clustered in the last three clusters. The five clustering centers of the width of the hilum plane are 89, 119, 144, 164, and 182, respectively, and most of the values are clustered in the latter four categories, that is, most of the values are more than 100. The five clustering centers of area are 6,602, 10,565, 15,656, 19,531, and 33,489, respectively, and most of the areas are clustered in the middle three categories, that is, the value is between 10,565 and 19,531. The five cluster centers of girth are 427, 600, 979, 2,811, and 5,553, respectively. Most of the values of the perimeter are clustered in the first two classes, indicating that most of the perimeter is less than 600. Through cluster analysis, the interpretability of data is increased. Hierarchical representation of the data of the four traits is more beneficial to establish the relationship between varieties and the prediction model of yield per unit area.

The HSV model is a color model similar to the way the human eye perceives color, which can intuitively express the light and shade, tone, and brightness of colors, facilitating color comparison and emotional expression. In this study, the lightness V in the HSV model is used to measure the seed coat glossiness. The conversion formula of the image from RGB color space to HSV color space is as follows:


(1) $$\left\{ \begin{array}{c} R^{\prime }=R/255\\ G^{\prime }=G/255\\ B^{\prime }=B/255 \end{array}\right.\,\begin{array}{c} \text{max}=\text{max}\,\left(R,G,B\right)\\ \min=\min\left(R,G,B\right) \end{array}$$

(2) H={0°,if⁢max=min60×°(G′-B′max-min+0),i⁢f⁢max=R′60×°(B′-R′max-min+2),i⁢f⁢max=G′60×°(R′-G′max-min+4),i⁢f⁢max=B′

(3) $$S=\left\{ \begin{array}{ccc} 0 & , & \text{max}=0\\ \frac{\text{max}-\text{min}}{\text{max}} & , & \text{max}\neq 0 \end{array}\right.$$

(4) V=max

The mean lightness of the whole seed region is calculated in the transformed HSV space as the final evaluation result of soybean seed glossiness. Seed coat uniformity reflects the uniformity of skin color distribution and is an important indicator to measure the quality of seeds. The method adopted in this study is to calculate the standard deviation δ from the RGB value of skin color in the whole seed region to represent the dispersion of seed skin color distribution. The formula is as follows:


(5) $$\delta =\sqrt{\frac{\sum_{\text{x}=0}^{\text{l}-1}\sum_{\text{y}=0}^{b-1}{\left[P\,\left(x,y\right)-\mu \right]}^{2}}{\text{A}}}$$

The L refers to the pixel length of soybean seed area, B refers to the pixel width of soybean seed area, A refers to the pixel area of soybean seed, P refers to the RGB value at the midpoint (X, Y) of seed image, and μ refers to the RGB mean value of seed coat area. The value of seed uniformity is between 0 and 1, and the closer the value is to 0, the more uniform the seed coat color distribution is. Due to the irregular traits of soybean seeds, it is difficult to measure the long and short axes directly. The length of the outer rectangle is the long axis of the soybean seed, and the width is the short axis of the soybean seed. According to the coordinate values of the four vertices of the outer rectangle, the specific pixel values of the long and short axes of soybean seeds are calculated. The girth of the hilum plane is obtained by calculating the number of pixels contained in the contour extracted from the edge of the seed image. The hilum is a strip on the concave side of soybean seed. It is a long, thin scar left after the seed has fallen off the seed stalk or placenta. It is round, oval, oval, etc. The color and shape of seed hilum of different soybean varieties are significantly different, so, the phenotypic traits of seed hilum could be used as an important parameter for seed identification. Seed kidney refers to the kidney-shaped region around the seed hilum, which is relatively dark in color and fuzzy in edge when observed by naked eyes. According to the experience of breeders, different varieties of soybean have significant differences in the performance of this part, so, the seed kidney is also used as an important phenotypic traits parameter to identify soybean seeds. The area of the hilum and kidney is very small, only relying on observation and measurement of the naked eye can’t be accurate and precise. With the aid of CV technology, we proposed an algorithm for identifying hilum and kidney based on edge detection, then, finally achieved the contour detection and segmentation of this narrow part and extracted the phenotypic traits parameters of this key part, which provided a new data reference index for soybean seed test and breeding program design. Gaussian Laplacian (LoG) operator is an edge detection operator based on the second-order differential method, which is composed of Gaussian filter and Laplace operator. The Laplace operator is used to extract the edge of the image by second-order differential operation, which can reflect the minimal change of gray value well and is suitable for edge detection with the small difference of gray value between seed hilum, seed kidney, and seed coat of soybean. In general, before edge detection, a two-dimensional Gaussian function is needed to denoise the image to improve the robustness of the operator to noise and discrete points, and then, edge detection is carried out. The point in the image whose second derivative is 0 is the target edge point. The two-dimensional Gaussian function can be expressed as follows:


(6) $$G\,\left(x,y\right)=\frac{1}{2\,\pi \,\sigma^{2}}\,{\text{e}}^{-\frac{{\text{x}}^{2}+{\text{y}}^{2}}{2\,\sigma^{2}}}$$

Let I (x, y) be the input image, then the calculation formula of edge detection by LoG operator is as follows:


(7) $$\hat{I}\,\left(x,y\right)=\nabla^{2}G\,\left(x,y\right)\,I\,\left(x,y\right)$$

**FIGURE 11:**
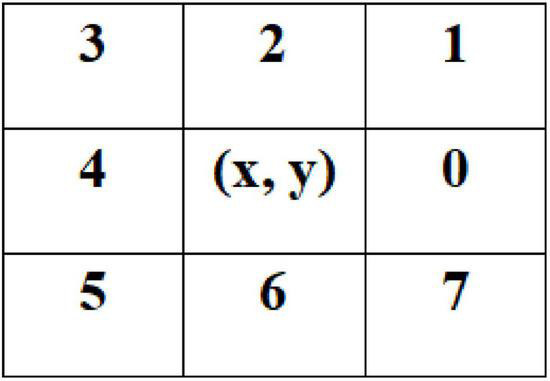
Pixel eight neighborhoods graph.

**FIGURE 12:**
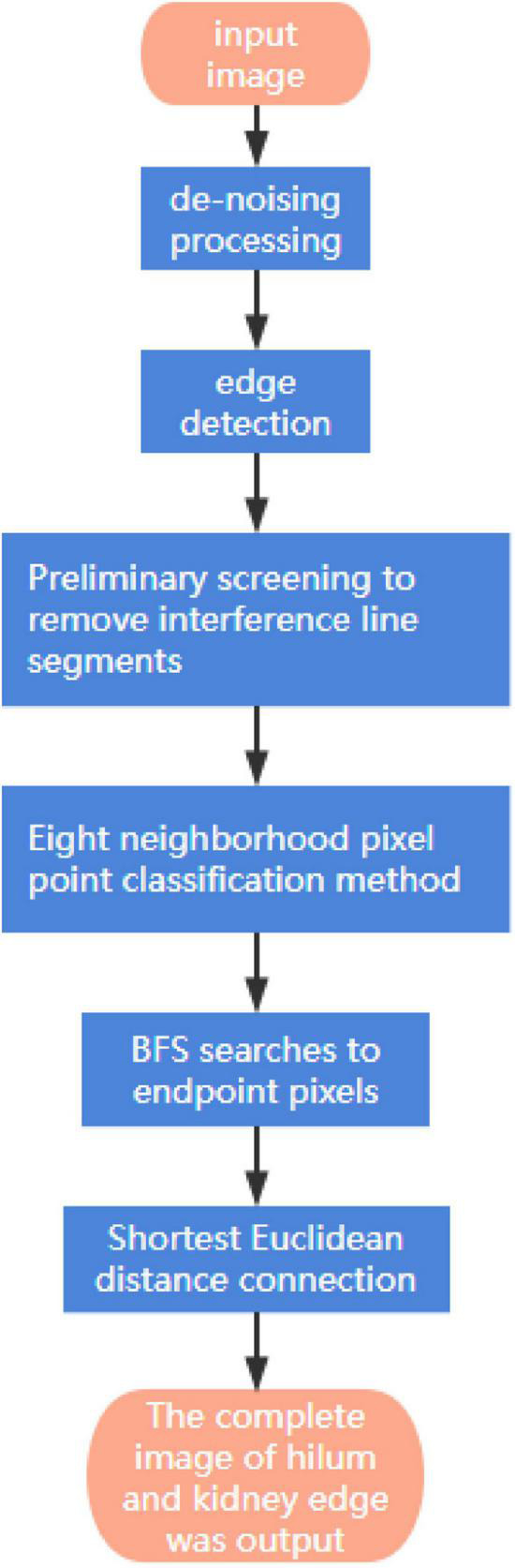
Edge contour algorithm steps.

When using the LoG operator to detect the edge of the hilum and kidney, there will be an edge breakpoint. Therefore, a method based on the breadth-first search is proposed to optimize the edge detection result of the LoG operator. The algorithm first classifies the detected target edge pixels to determine whether it is an endpoint; the method is to search the eight neighborhood pixels around the pixel. If only one of the eight neighborhood pixels is connected to the pixel, the pixel is an endpoint, and the endpoint is saved in the endpoint array, otherwise, the pixel is a non-endpoint. For the non-endpoint, if there is no pixel connected to it in the neighborhood, it is an isolated point. A point is on the edge-line if two endpoints are connected to it in a neighborhood of eight; If there are four points connected to it in the neighborhood, it is an edge-line crossing point. The eight-neighborhood map of pixels is shown in Figure 11. Then, the breadth-first algorithm is used to search the pixels in the endpoint array until the search stop condition is met, and the search path is recorded. Then, the endpoint with the shortest Euclidian distance is selected from the breakpoint to connect, and the complete hilum and kidney edges are obtained. In the actual process, it is necessary to screen the edge segments searched, remove the interference segments that do not belong to the seed hilum and seed kidney parts, then, connect the edge segments that meet the requirements, and finally, obtain the complete edge contour of soybean seed hilum and seed kidney. The specific steps of the algorithm are shown in Figure 12.

## Database and Soybean Visual Artificial Intelligence Breeding Platform

**FIGURE 13:**
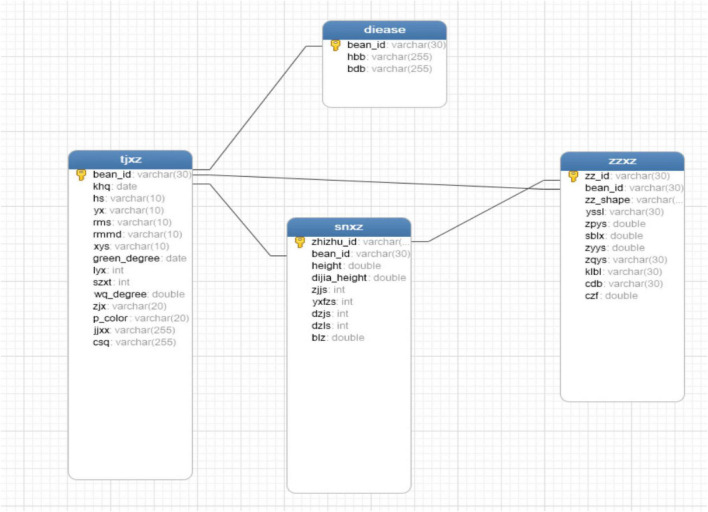
Original ER diagram.

**FIGURE 14:**
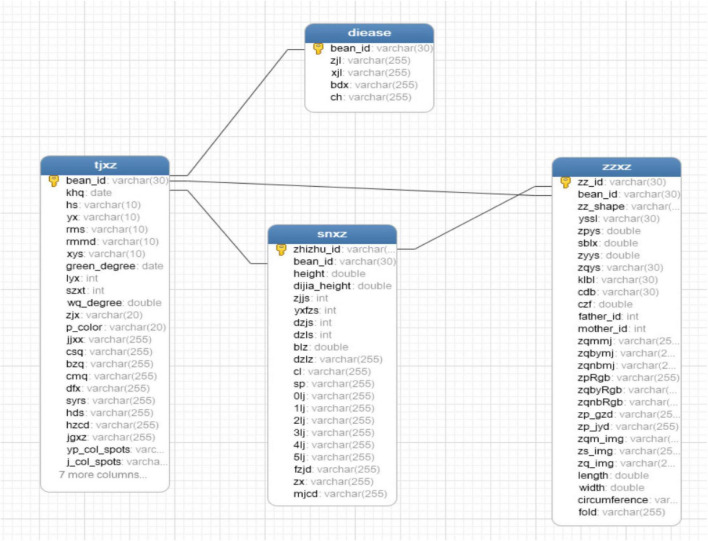
Extended ER graph.

Systematic collection, management, and analysis of phenotypic data have shown certain genetic gains in accelerated breeding (Araus et al., 2018). Therefore, the construction of a phenotypic database is an essential step. The database entity relationship (ER) diagram corresponding to various original traits is shown in Figure 13, and the database ER diagram corresponding to expanded traits is shown in Figure 14.

The database structure and relationship corresponding to original traits are shown in Figure 13, which only included field traits table (tjxz), disease traits table (disease), and indoor traits table (snxz). The field traits table includes variety number (bean_id), flowering period (khq), colors of flowers (hs), shapes of leaf (yx), fluffy color (rms), density of trichome (rmmd), number of lobules (xys), green degree of leaves (green_degree), deciduous (lyx), growth form (szxt), pod bending degree (wq_degree), blasting pods (zjx), pod color (p_color), pod bearing habit (jjxx), and mature period (csq), with a total of 15 fields. The disease traits table contains three fields, including variety number, gray spot (hbb), and virus disease (bdb). The indoor traits table included plant number (zhizhu_id), variety number (bean_id), plant height (height), base pod height (dijia_height), number of the nodes on main stem (zjjs), effective branch number (yxfzs), pods per plant (dzjs), number of grains per plant (dzls), and 100-grain weight (blz), with a total of 9 fields. The soybean seed traits table (zzxz) includes seed number (zz_id), variety number (bean_id), seed shape (zz_shape), seed coat color quantity (yssl), seed coat color (zpys), seed coat spot type (sblx), cotyledon color (zyys), seed hilum color (zqys), seed coat cracking ratio (klbl), seed coat luster (zpgz), seed crude protein content (cdb), and seed crude fat content (czf). In the four tables, the plant number (zhizhu_id) of the indoor traits table (snxz) is used as both a primary key and a foreign key, which is associated with the seed number (zz_id) of the seed traits table. The variety number (bean_id) is used as the foreign key to be associated with the primary key variety number (bean_id) of the field traits table (tjxz). The variety number (bean_id) of the disease traits table (disease) is both a primary key and a foreign key, which is associated with the various number (bean_id) of the field traits table. The seed traits table (zzxz) is a table, in which the seed number (zz_id) acts as the primary key, the variety number (bean_id) serves as a foreign key in the table and is associated with the primary key in the field traits table.

The structure and relationship of database tables corresponding to existing traits are shown in Figure 14, including field traits table (tjxz), disease traits table (diease), indoor traits table (snxz), and seed traits table (zzxz). The expanded field traits table (tjxz) includes variety number (bean_id), flowering period (khq), colors of flowers (hs), shapes of leaf (yx), fluffy color (rms), density of trichome (rmmd), number of lobules (xys), green degree of leaves (green_degree), deciduous (lyx), growth form (szxt), pod bending degree (wq_degree), blasting pods (zjx), pod color (p_color), pod bearing habit (jjxx), mature period (csq), sowing date (bzq), emergence date (cmq), lodging (dfx), days of growth period (syrs), number of flowers (hds), rachis length (hzcd), pod shape (jgxz), color and spots of leaf (yp_col_spots), color and spots of stem (j_col_spots), field distribution photographs (sunny day) (s_pho), field distribution photographs (overcast) (c_pho), field plant coverage (fgl), canopy density (ybd), amount of weeds (zcl), precipitation during flowering period (kh_jsl), and plant density (density). The expanded indoor traits table (snxz) includes plant number (zhizhu_id), variety number (bean_id), plant height (height), base pod height (dijia_height), number of the nodes on main stem (zjjs), effective branch number (yxfzs), pods per plant (dzjs), number of grains per plant (dzls), 100-grain weight (blz), grain weight per plant (dzlz), yield (cl), rotation video of single plant (sp), pod with 0 bean (0lj), pod with 1 bean (1lj), pod with 2 beans (2lj), pod with 3 beans (3lj), pod with 4 beans (4lj), pod with 5 beans (5lj), branching angle (fzjd), plant shape (zx), and length of each section (mjcd). The expanded disease traits table (diease) includes variety number (bean_id), fungal disease (zjl), bacterial disease (xjl), viral disease (bdx), and insect pest (ch). The expanded soybean seed traits table (zzxz) includes seed number (zz_id), variety number (bean_id), seed shape (zzxz), seed coat color quantity (yssl), seed coat color (zpys), seed coat spot type (sblx), cotyledon color (zyys), seed hilum color (zqys), seed coat cracking ratio (klbl), seed coat luster (zpgz), seed crude protein content (cdb), seed crude fat content (czf), male parent number (father_id), female parent number (mother_id), area of hilum plane (zqmmj), the area of the marginal part of the hilum (zqbymj), the area inside the hilum (zqnbmj), seed coat RGB (zpRgb), hilum margin position RGB (zqbyRgb), inside hilum RGB (zqnbRgb), seed coat glossiness (zp_gzd), seed coat uniformity (zp_jyd), photograph of hilum plane (zqm_img), photograph of kidney (zs_img), photograph of hilum (zq_img), length of hilum plane (length), width of hilum plane (width), girth of hilum plane (circumference), and degree of seed folding (fold). The relationship between the four tables after expansion is the same as before.

In the original table structure, there is a lack of phenotypic trait information, and the disease table could only store the information of two diseases, and the correlation between the four tables is low. In the expanded table structure, the gaps of seed phenotypic traits are filled in, and the disease traits table could store four kinds of disease information, enrich the fields of each table, and increase the correlation among the tables. Among them, if the plant number in the indoor traits table is the same as the seed number in the seed traits table, it means that the phenotypic information of the plant is the corresponding phenotypic information of the seed when it grows into a plant, thus, associating the information of two stages of the life of the same soybean.

Firstly, the field information of each variety is obtained through mobile phone terminal and CV technology, and the field information of each variety is stored in the field traits table. Then, parts of the collected soybean disease images are used for deep learning training, so that the trained model can recognize each disease. The model is used to identify the disease images of different varieties, and the disease image information and disease category of different varieties are saved in the disease traits table. Variety numbers and various information of all varieties are included in the field trait table. The variety number in the disease traits table is restricted by the variety number in the field traits table. Before planting, the soybean is fixed on a specific device for image collection, and the data of various phenotypic traits of seeds are obtained by using computer image processing technology and CV technology, and all seed data are imported into the seed traits table in batches. After the plants corresponding to the seeds are harvested, the plants are fixed on a specific rotating device in the indoor space for 360° video collection. Images of specific frames are captured in the video to label pods with each grain number, and then, the improved YOLOV4 algorithm mentioned above is used to train the model. The trained model could recognize several pods, and the number of pods, pods per plant, and seeds per plant could also be obtained. The phenotypic information of base pod height and plant height is also obtained by CV technology after capturing specific frames of images. Finally, the obtained plant information is imported into the indoor traits table in batches. The phenotypic information of a seed is associated with the corresponding plant information by numbering. Variety numbers of indoor and seed traits table are constrained by variety numbers of field traits table.

**FIGURE 15:**
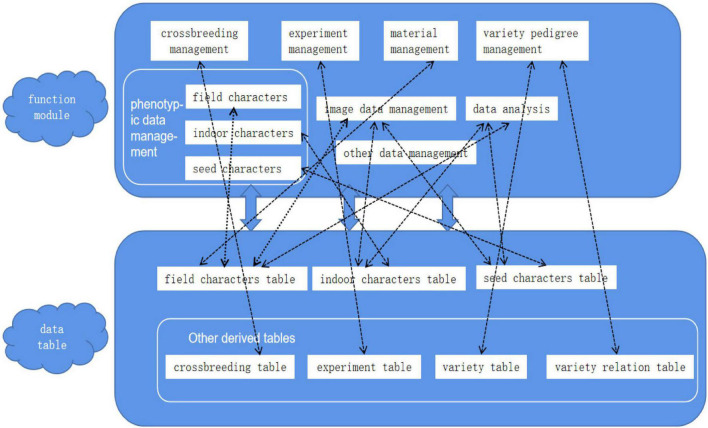
Function module—data table.

The soybean visual AI breeding platform corresponding to the database system is divided into 8 functional modules, which are crossbreeding management, experiment management, material management, variety pedigree management, phenotypic data management, image data management, data analysis, and other data management. Phenotypic data management is divided into field traits, indoor traits, and seed traits. The data tables in the ER diagram above and derived data tables together constitute a complete database system, and each table is closely linked with functional modules, as shown in Figure 15. The close connection of the data table, and the forming of the functional correlation, so that the phenotypic information of the seed, the phenotypic information of the plant corresponding to the seed, and the phenotypic information of the variety to which the seed belongs can be queried at a time, bringing the previously independent loose data closer together. Some fields in each table can also be input into the SVM model as predictors for training, and the final model can predict the category of a certain trait. The filtering of fields as predictors and the determination of target fields are the focus of subsequent work. When the amount of data reaches a certain level, big data technology can be used to mine and analyze the data of each field.

## Conclusion

In this study, three types of traits including field traits, indoor test traits, and disease traits are expanded. At the same time, soybean seed traits are added, and the role of the expanded phenotypic traits is explained. It is convenient to use CV technology to quickly obtain data and then, perform machine learning, which can provide big data support for breeding experts to design accurate breeding programs.

The CV test has a greater advantage over the traditional manual test and the earlier CV test. For the extraction of phenotypic traits such as plant height and base pod height, the error is within one pixel, that is, the accuracy reaches millimeter level. The image processing program processes images in batches. Compared with the traditional manual test, the data collection speed is greatly improved, and it has high throughput. The area of each part of the extracted soybean seeds and the accuracy of the length and width data of the seeds have also reached the millimeter level. These phenotypic data cannot be obtained by traditional artificial seed testing.

The improved YoloV4 algorithm training model is used to detect the pods of plants. At present, the average recognition accuracy of various types of pods is about 84.37%, and the average recognition accuracy of one to four pods has reached more than 90%. The model is still being further tuned and tuned. There are large number of pods on a single soybean plant and many pods shade each other. Manual observation will inevitably cause errors and consume a lot of time. This method is more accurate and efficient to obtain the data of the number of each pod and completed tasks that are difficult for traditional manual test species. Traditional plant phenotypic information acquisition mainly relies on manual measurement, naked eye observation, and software analysis after image acquisition. The grain size of soybean seeds is small and contains multiple phenotypic traits, so, it is difficult to obtain phenotypic data by manual measurement and naked eye observation, and some image analysis software is not flexible enough to meet the needs of breeders to obtain all the required phenotypic traits. By using CV and machine learning technology, soybean phenotypic traits can be acquired more accurately and efficiently. Compared with traditional methods, phenotypic traits are obtained more comprehensively. This method is a supplement to current plant Phenomic methods. Traditional methods can be used to validate the data acquired by machine learning. The phenotypic traits of a soybean are the physical expression of its genetic genes. More data of phenotypic traits are extracted, which could be used to establish the prediction model of soybean yield per unit area, and to provide data support for establishing the relationship between phenotypic traits and genome.

All traits of soybean directly or indirectly affect breeding. Many studies have shown that some phenotypic traits directly affect soybean yield. The phenotypic traits of a soybean are all intuitive, visible, and easy to measure, but the deeper level of the soybean gene remains to be studied (Cheng et al., 2016). Propose a method for rapid extraction of soybean seed DNA with a success rate of more than 98%, which can be used for genetic analysis. With the further application of technology in seed tests, CV technology can obtain more or more accurate traits, which can provide more big data support for breeding experts to optimize breeding programs. The problem we will solve is not only to establish the relationship between phenotypic traits and genomes, and the relationship between soil and ecological environment of crop growth, but also to establish a soybean big data platform, providing breeding experts with big data services in an easy-to-understand way of knowledge expression.

## Data Availability Statement

The raw data supporting the conclusions of this article will be made available by the authors, without undue reservation.

## Author Contributions

YX contributed to the research of data expansion and the writing of the article. PL contributed to the pest part. HH contributed to the conception and modification of the article. JL and HY provided guidance for professional knowledge. XF contributed to the modification of the article. All authors contributed to the article and approved the submitted version.

## Conflict of Interest

The authors declare that the research was conducted in the absence of any commercial or financial relationships that could be construed as a potential conflict of interest.

## Publisher’s Note

All claims expressed in this article are solely those of the authors and do not necessarily represent those of their affiliated organizations, or those of the publisher, the editors and the reviewers. Any product that may be evaluated in this article, or claim that may be made by its manufacturer, is not guaranteed or endorsed by the publisher.
